# The rocks and hard places of MAiD: a qualitative study of nursing practice in the context of legislated assisted death

**DOI:** 10.1186/s12912-020-0404-5

**Published:** 2020-02-17

**Authors:** Barbara Pesut, Sally Thorne, Catharine J. Schiller, Madeleine Greig, Josette Roussel

**Affiliations:** 1 Canada Research Chair in Health, Ethics, and Diversity, University of British Columbia Okanagan, 1147 Research Road, Okanagan, Kelowna, BC V1V 1V7 Canada; 2 University of British Columbia, T201-2211 Wesbrook Mall, Vancouver, BC V6T 2B5 Canada; 3 University of Northern British Columbia, Prince George, BC V2N 4Z9 Canada; 4 Policy, Advocacy and Strategy, Canadian Nurses Association, 50 Driveway, Ottawa, Ontario K2P 1E2 Canada

**Keywords:** (3-10) Assisted death, Medical assistance in dying, Euthanasia, Palliative care, Nursing practice, Assisted suicide, Legislation

## Abstract

**Background:**

Medical Assistance in Dying (MAiD) was legalized in Canada in June, 2016. The Canadian government’s decision to legislate assisted dying, an approach that requires a high degree of obligation, precision, and delegation, has resulted in unique challenges for health care and for nursing practice. The purpose of this study was to better understand the implications of a legislated approach to assisted death for nurses’ experiences and nursing practice.

**Methods:**

The study used a qualitative approach guided by Interpretive Description. Semi-structured interviews were conducted with 59 registered nurses and nurse practitioners. Interviews were audio-recorded, transcribed, and managed using qualitative analysis software. Analysis followed a procedure of data immersion, open coding, constant comparative analysis, and the construction of a thematic and interpretive account.

**Results:**

Nurses in this study described great variability in how MAiD had been enacted in their work context and the practice supports available to guide their practice. The development of systems to support MAiD, or lack thereof, was largely driven by persons in influential leadership positions. Workplaces that supported a range of nurses’ moral responses to MAiD were most effective in supporting nurses’ well-being during this impactful change in practice. Participants cited the importance of teamwork in providing high quality MAiD-related care; although, many worked without the benefit of a team. Nursing work related to MAiD was highly complex, largely because of the need for patient-centered care in systems that were not always organized to support such care. In the absence of adequate practice supports, some nurses were choosing to limit their involvement in MAiD.

**Conclusions:**

Data obtained in this study suggested that some workplace contexts still lack the necessary supports for nurses to confidently meet the precision required of a legislated approach to MAiD. Without accessible palliative care, sufficient providers, a supportive team, practice supports, and a context that allowed nurses to have a range of responses to MAiD, nurses felt they were legally and morally at risk. Nurses seeking to provide the compassionate care consistent with such a momentous moment in patients’ lives, without suitable supports, find themselves caught between the proverbial rock and hard place.

## Background

All forms of assisted suicide were illegal in Canada until February 2015 when the Supreme Court of Canada (SSC) released its landmark decision *Carter v Canada (Attorney General)* (“*Carter*”) [1]*.* In its ruling, the SCC struck down the *Criminal Code’s* prohibition on assisted suicide for competent adults in certain clinical circumstances, on the basis that such a prohibition unjustifiably violated the *Canadian Charter of Rights and Freedoms* (“*Charter*”).

The SCC’s ruling gave the federal government time to craft a legislative framework to regulate assisted dying. In June 2016, medical assistance in dying (MAiD) was legalized in *An Act to amend the Criminal Code and to make related amendments to other Acts (medical assistance in dying)*, a statute still colloquially known as *Bill C-14* [2]. The government had crafted a new concept, MAiD, within this legislation rather than continue to use an existing term, such as “physician assisted suicide” or “physician assisted dying”. This new terminology represented a recognition that a team of healthcare providers, not only physicians, is typically required to implement such a complex procedure [3]. In *Bill C-14*, MAiD is defined as: a) the administration by a medical practitioner or nurse practitioner of a substance to a person, at their request, that causes their death; or b) the prescribing or providing by a medical practitioner or nurse practitioner of a substance to a person, at their request, so that they may self-administer the substance and in doing so cause their own death Only 6 of the 6749 medically assisted deaths recorded in Canada between December 10, 2015 and October 31, 2018 were self-administered [4].

According to *Bill C-14*, to be eligible for MAiD, an individual must meet all of the following criteria: (a) they are eligible for health services funded by a government in Canada; (b) they are at least 18 years of age and capable of making decisions with respect to their health; (c) they are suffering from a grievous and irremediable medical condition; d) they have made a voluntary request for medical assistance in dying that was not made as a result of external pressure; and (e) they give informed consent to receive medical assistance in dying after having been informed of the means that are available to relieve their suffering, including palliative care [2]. Once *Bill C-14* was passed, provincial and territorial governments, as well as provincial and territorial regulatory bodies for the health professions, became responsible for enacting policies, procedures and processes to guide MAiD-related healthcare practice in Canada.

### Implications of a legislated approach to MAiD

The Canadian government chose to enact legislation that would regulate assisted suicide, but there were other options available to them. Luzon modeled five approaches to assisted death based upon obligations, precision, and delegation:

*Obligation means that people are legally bound by a rule, so that their behavior is subject to examination under the general rules, procedures, and discourse of the law. Precision means that rules unequivocally define the conduct they require, authorize, permit, or prohibit. Delegation refers to the body that has been granted authority (by the public) to determine, implement, interpret, and apply the rules. All three dimensions can vary in degree. Based on these characteristics, legalization may be* ***hard*** *(where all three properties are maximized),* ***soft*** *(where some properties are maximized and others minimized), and* ***null*** *(where all three properties are minimized).* [emphasis added] (5 p. 7)

The five possible legal framework responses to assisted death are as follows. The first would involve maintaining the *status quo*, such that assisted death would continue to be treated as a crime; this would have been an obviously problematic approach given the existence of the *Carter* decision. The second is *defense* in which it would be recognized that there may, at times, be situations in which a valid defense to assisted death can be made. The third involves *de-prioritization*; this response would allow laws against assisted suicide to remain in place but it would not be viewed as a priority of the justice system to prosecute those who are involved in assisted dying or to impose criminal sanctions upon them. The fourth is *de-criminalization* in which no precise laws are provided; this was the approach used by the Canadian government after the *Criminal Code* prohibition on abortion was struck down by the SCC in the 1988 *R v Morgentaler* case. The fifth is *legislation* in which “*there is a specific binding law (high in obligation), a precise, specific, clear rule for every practice (high in precision), and the designated third party to which the state delegates authority is the legislature (high in delegation).*” (5 p. 14)

Canada chose the fifth approach to assisted death which, according to Luzon, entails a ***hard*** approach characterized by a high degree of obligation, precision, and delegation [5]. To that end, *Bill C-14* incorporated numerous safeguards and requirements into the MAiD process. For example, eligibility for MAiD must be determined by two practitioners, either physicians or nurse practitioners, who are independent of one another (the second practitioner must also be independent of the patient). Once the patient has been determined to be eligible, he or she must then submit a written request for MAiD in the presence of two independent witnesses. In addition, there is a mandatory reflection period of at least 10 days between the signing of that written request by the patient and the day that MAiD is actually provided, although this can be shortened in certain clinical circumstances [2]. These, and other, safeguards were written into the legislation by the government to decrease the possibility that the MAiD procedure could be used inappropriately.

### The nursing role in MAiD

Not only has Canada taken the ‘hardest’ approach to assisted death, but it is also the first country to allow nurse practitioners to act as MAiD assessors and providers. Although it is important to note that this role for nurse practitioners is further regulated at the provincial level and so not all nurse practitioners are allowed by the provincial health regions to act as MAiD assessors or providers. In Canada, registered nurses who do not hold a nurse practitioner credential also play important roles in MAiD. The important role of the registered nurse is also evident in studies from other countries where assisted death is legal [6–11]. For example, our synthesis of qualitative studies from Belgium, the Netherlands, and Canada of registered nurses’ experiences with assisted death suggested that nurses perform a central role in negotiating initial inquiries about assisted death, that nurses provide important ‘wrap-around’ care for patients and family, and that participating in an assisted death was impactful for nurses and required significant moral work [12].

In consideration of the importance of the registered nursing role, and the new role for nurse practitioners in Canada, we conducted a study in which we explored the policy, practice, and ethical implications of MAiD for nursing. This was a two-phased study in which we first conducted systematic reviews of the literature [12–14] and then a qualitative study of Canadian nurses’ experiences with MAiD. As part of the literature synthesis we gathered and analyzed nursing regulatory documents that were created to guide nursing practice in MAiD from the 10 provinces and 3 territories in Canada [13]. We discovered substantial variability in the degree to which these regulatory bodies chose to provide additional guidelines for nurses beyond what was provided in the MAiD legislation. As such, we were interested in better understanding how nurses were experiencing the enactment of the legislation in their practice related to MAiD, and thus explored this qualitatively. In this paper, we report on findings from the qualitative phase of the study that revealed the impact of Canada’s legislated approach to assisted death on nurses’ experiences, and on nursing practice, in Canada.

## Methods

This qualitative study was guided by Interpretive Description, a pragmatic approach to developing knowledge for a practice discipline [15].

### Participants

Data was collected through 60 interviews with 59 participants (see Table 1 for demographic data). Recruitment of this sample occurred via bulletins that were distributed to key stakeholders and prospective participants using convenience, purposive, and snowball sampling techniques. For example, we advertised through the Canadian Nurses’ Association, through health regions, and through the Canadian Association of MAiD Assessors and Providers. We asked interview participants to pass the study information on to others. We sought to gain participation from all English-speaking provinces. We did not specifically target the Canadian territories as the interim reports on MAiD produced by Health Canada suggested that few cases were occurring in those areas [4]. Eligibility criteria required that participants were registered nurses or nurse practitioners, who had previously cared for patients requesting or receiving MAiD, or those registered nurses or nurse practitioners who had decided, for whatever reason, not to participate in the MAiD process. No participants dropped out of the study or requested that their data be removed from the study. These 59 participants had significant experience with MAiD. For example, 24 of the 59 participants had conducted more than 25 conversations with patients about MAiD, and 11 of the 59 participants had been involved with more than 25 patients who went on to receive MAiD.

**Table 1:** Demographics of Study Participants

Characteristic	Number of Participants *n* = 59
Province	British Columbia: *n* = 28 (48%) Ontario: *n* = 16 (27%) Manitoba: *n* = 7 (12%) Alberta: *n* = 5 (9%) Newfoundland and Labrador: *n* = 2 (3%) Saskatchewan: *n* = 1 (2%)
Age	25–44: *n* = 27 (46%) 45–64: *n* = 29 (49%) > 65: *n* = 3 (5%)
Gender	Female: *n* = 56 (95%) Male: *n* = 3 (5%)
Ethnicity	Caucasian: *n* = 57 (97%) Other: *n* = 2 (3%)
Designation	Registered Nurse: *n* = 43 (73%) Nurse Practitioner: *n* = 13 (22%) Clinical Nurse Specialist: *n* = 3 (5%)
Years Worked	2–4 years: *n* = 4 (7%) 5–9 years: *n* = 10 (17%) 10–14 years: *n* = 13 (22%) 15–19 years: *n* = 4 (7%) 20–24 years: *n* = 6 (10%) > 25 years: *n* = 22 (38%)
Work Context	Home & Community: *n* = 32 (54%) Acute Care: *n* = 10 (17%) LTC: *n* = 5 (9%) Hospice: *n* = 4 (7%) Clinic: *n* = 3 (5%) Other: n = 5 (9%)
Conscientious Objection	No/Unsure: *n* = 50 (85%) Yes: *n* = 9 (15%)
Spiritual or Religious Affiliation	Religious or Spiritual: *n* = 33 (56%) Neither: *n* = 15 (25%) Spiritual but not Religious: *n* = 11 (19%)

### Data collection and analysis

Data was collected in the fall of 2018 and the spring of 2019, approximately 2 years after the MAiD legislation was enacted. Semi-structured interviews, conducted by telephone, were used to garner an in-depth understanding of nurses’ experiences with MAiD. Telephone interviews were necessary to reach nurses from across Canada. Interviews were conducted by the principal investigator and research coordinator. Participants were provided with a detailed consent form at least 24 h prior to the interview to ensure that they understood the focus and objectives of the study. Interviews were conducted only after the signed consent was received. Interviewers reiterated the rationale for conducting the study prior to the interview, and participants were provided with an opportunity to ask questions. A semi-structured interview guide was developed for this study, piloted and refined prior to data collection (Additional file 1). Examples of interview questions included: (i) Can you tell us how the process of MAiD occurs in your practice context? (ii) What resources and practice supports are available to assist you in caring for MAiD patients? (iii) Tell us about your experiences with MAiD? The average length of interviews was 55 min. In totality, 2992 min of interview data were collected and subsequently analyzed.

Interview data were audio-recorded, transcribed verbatim, de-identified, checked for accuracy and uploaded into NVivo^12TSN^ for data analysis and management. Transcripts included emotions evident during the interview (e.g., crying). All audio recordings were reviewed by the principal investigator and detailed field notes were written and referred back to during the analytic process. Data were analyzed following the logic of Interpretive Description [15]. Open codes were developed and negotiated by two investigators (BP & MG) after an immersion process of reading and re-reading multiple transcripts. These codes were further refined with input from two additional investigators (ST & JR). These open codes were then used to code the remaining data. Codes were further refined in an iterative process of data collection and analysis by using constant comparative data analysis techniques, a technique developed initially within Grounded Theory [16]. Once all of the transcripts had been coded, data contained within these codes were summarized to construct a thematic and interpretive account of Canadian nurses’ experiences with MAiD. In this paper we discuss the experiences related to Canada’s legislated approach to MAiD.

## Results

Nurses interviewed for this study described great variability in how MAiD had been enacted within their geographic and work context and how that variability had influenced their experiences with MAiD. This variability was largely influenced by three themes: (1) the leadership taken by influential persons within systems, (2) the presence and nature of a multi-disciplinary team, and (3) the systems’ complexity and capacity to support MAiD.

### Systems: influential leaders setting the tone

Nurses described work contexts that ranged from a virtual absence of any MAiD-related guidelines to highly structured systems in which a comprehensive set of supports existed to guide nursing practice. In some contexts, policies and procedures were established fairly quickly. For example, one participant described how, after the legislation was passed, key leaders in the health region immediately established a working group to work intensively over a weekend to construct the policies and procedures that would guide immediate practice. However, in other contexts those first MAiD cases were done with little direction, “*We really had no idea what we were doing because we hadn’t actually made any policy or guidelines yet*.” P42 Even many months after the legislation, some nurses were still working within a healthcare policy and procedure void. These findings were similar for both registered nurses and nurse practitioners, although nurse practitioners had the structure provided for them within *Bill C-14*. Nurses, however, sometimes found themselves trying to assist in a MAiD procedure with no practice guidelines in their places of work. This created uncertainty in their practice, particularly when nurses remained the primary caregivers of patients contemplating or undergoing MAiD, which also involved high levels of interaction with their families. “*So, my big concern is if someone does approach me with a written request, what do I do from there? And I know the health region has developed no policies pertaining to what the process is*.” P26.

Much of this variability in the degree of practice support was a result of the decisions (or lack of decisions) made by persons in influential leadership positions either immediately preceding or following the legalization of MAiD. For example, one participant mentioned that a change in government soon after the *Carter* decision had slowed down the development of MAiD guidelines in their province which in turn heighted the perception of risk. “*With the change in government, things were a little bit stalled and questions weren’t necessarily being answered. I think there were a lot of physicians and NPs maybe a little bit nervous about how things were working*.” P1 Some health authorities assigned key individuals to lead the development of practice supports. A number of innovations were developed, including regional interdisciplinary MAiD teams, designated persons to work alongside and support individuals considering whether to undergo MAiD, and therapeutic interventions designed to address the underlying suffering that had contributed to a MAiD request. However, leaders also developed organically as they championed the MAiD process. For example, this nurse participated in a provision while her clinical leader was away. “*Because she was away, I informally became the leader of MAiD on our unit*.” P50.

Leaders responsible for palliative care were particularly influential in the development of structures and processes to support MAiD. The beliefs of these leaders about the acceptability of MAiD, its fit with palliative care, and perhaps most importantly, their recognition that MAiD would generate a range of moral responses in their colleagues, determined the direction and outcomes of these practice supports. For example, in one jurisdiction, MAiD assessment responsibilities were assigned to nurse practitioners who were engaged in palliative care. This decision meant that palliative care and MAiD were both integrated within nursing responsibilities. As logical as this decision seemed from a workflow perspective, it resulted in unique tensions, particularly for those palliative nurses who objected to MAiD due to either their moral values or their beliefs about its fit with a palliative care philosophy.

Another example of influence was how leaders constructed workplace policies to support a range of moral responses to MAiD. We specifically use the term ‘range of moral responses,’ rather than conscientious objection, to reflect the uncertainty about MAiD that was characteristic of nurses in this study. Few openly declared themselves as conscientious objectors; instead, more were uncertain about how they felt about MAiD. Nurses described workplace policies that varied dramatically in how they accommodated their nurses’ willingness, or not, to participate in MAiD and the uncertainty that caused. At one end of the spectrum, nurses were allowed to take a day off without pay if they were uncomfortable with MAiD and it was occurring on their unit. At the other end of the spectrum, nurses were expected to provide all non-MAiD related care, no matter how they felt about MAiD. These policies reflected very different approaches to nurses’ moral well-being. As employees of healthcare, nurses felt they had little control over the ways in which their workplaces were structured to accommodate their comfort level with MAiD. For example, one nurse stated, “*we need some sort of support groups or guidelines for conscientious objectors. I have heard that other countries have more lenient processes for conscientious objectors so that they don’t feel stigmatized*.” P54.

Nurses perceived physicians, and in particular palliative physicians, to be important influencers in how MAiD processes developed. For example, one participant described how the medical director influenced the implementation of MAiD on her palliative unit. “*Our medical director at the time wasn’t on board and that trickled down to all of us*.” P57 Unlike nurses who were employees of healthcare, physicians were perceived as having more latitude to choose whether, how, and to what extent they would support the MAiD process. In some cases, nurses described how physicians worked with them to seamlessly integrate MAiD with palliative care. In other cases, nurses described physicians who erected barriers to patient involvement with MAiD. These barriers could include telling patients that they were not quite ready for MAiD, or suggesting that it could not be done in the community where patients were living, or simply ignoring patient requests. The strongest type of physician resistance described by participants was the withdrawal of palliative care services once a patient had chosen MAiD. This withdrawal of services made it difficult for nurses to support good pain and symptom management while the patient was awaiting MAiD. Nurses responded strongly to this withdrawal of palliative services: “*So we have a serious practice issue here. I’m mad as hell.”* P24.

However, participants also suggested that relationships between those who provided palliative care and those who provided MAiD were becoming more congenial over time. “*We have come a long way because people are not so angry or defensive.”* P31 In some cases, this was because MAiD teams had been formed outside of palliative care teams and they had learned to work together. In other cases, palliative clinicians were becoming more comfortable with MAiD as an option, either within or outside of palliative care. Despite this apparent easing of relationships between MAiD and palliative providers, there remained significant concerns related to the inadequacy of palliative care systems in Canada and the impact that MAiD could have in the face of this inadequacy. For example, one participant suggested that the workload generated by MAiD could be a significant barrier for palliative care clinicians who were already working to maximum capacity. Another participant suggested that palliative care, which already carried a fair bit of stigma because of its relationship to death, would become further stigmatized with the introduction of MAiD. Ultimately, this perception would lead to even less acceptability and uptake of palliative care by patients. But what created wider concern for nurses was the inadequate accessibility to palliative care services for some patients in Canada. Under *Bill C-14*, clinicians are required to offer palliative care to clients who are considering a MAiD death. Participants reflected on the irony of how much attention had gone into supporting accessibility to MAiD without corresponding attention paid to overall accessibility to palliative care. “*We use this rhetoric that it’s somebody’s right to die and I don’t want to debate that part but I think it’s also their right to have access to care done by clinicians who are knowledgeable about palliative care.”* P23 This participant was reflecting on the paucity of specialized palliative care but also on the lack of palliative care knowledge within primary care where most palliative care happens. This same participant went on to describe how providers’ lack of palliative care knowledge unwittingly contributed to patient suffering. This tension between a system that caused undue suffering because of ignorance of good palliative care, and a system designed to relieve that suffering through MAiD, put this nurse in an intense state of tension:

*This is the crazy thing for me to consider … it's a shame and it's something that I grieve to think that our system, as it is, can contribute so much to the suffering of somebody on so many different levels. … on top of whatever illness process that is causing suffering. But that our health care system contributes to suffering, and is doing nothing about our own contribution to that suffering, but then uses that very suffering to activate access to MAiD. It's absolutely ridiculous to me. P23*

In light of the tensions between palliative care and MAiD, participants had thoughtfully considered what they thought might be the ideal relationship between the two systems. One participant described it as “*parallel lines with crossover points*.” P24 MAiD providers would work along one continuum while palliative care providers would work along the other continuum. But, if and when a client should choose to cross over from palliative care to MAiD, then palliative care would continue as an unbroken commitment to patients.

In summary, nurses in this study were working within systems that differed greatly in their response to MAiD. Some were highly organized whereas others were devoid of policies, procedures, and formal direction. Much of this variability was attributed to the way in which influential leaders, particularly those with responsibilities for palliative care, had chosen to approach MAiD. Further, perspectives of these influential palliative leaders had in turn been influenced by the broader challenges of palliative care accessibility in the Canadian context.

### Teamwork: Two’s a team

Nurses in this study participated in MAiD teams to varying degrees. At one end of the spectrum, nurses worked in isolation, being lone assessors and/or providers who worked only peripherally with other assessors and providers. At the other end of the spectrum were nurses who were integrated into well-connected teams dedicated to providing MAiD. In the middle were nurses who worked organically and closely with a few physicians but who were outside of a formal team structure. Even as they found themselves with varying degrees of team support, participants described teamwork as essential to a successful MAiD process. MAiD was a new procedure, and participants described the time it took for physicians and nurses to create a MAiD process that worked well and to feel comfortable with that process. Nurses suggested that, at minimum, two people should be present at every provision of MAiD, one to do the provision and one to look after family and friends and to troubleshoot situations that arose during the process. Having a second person was particularly important in light of the impactful nature of the experience and the need to ensure a seamless, trouble-free provision. For example, this nurse talked about a difficult provision and the importance of a supportive physician. “*It was just me, the doctor and the patient and it was a bad feeling, dark, no windows. Afterward I had to wait for the funeral home and I said to the physician, ‘you can go.’ He said, ‘I’m not leaving you.’ So, it was just so nice to have that support from the physician*.” P37 As physicians were often “*piloted in*” to perform the procedure, it was the nurses who ultimately learned what worked well and who were often in a position to provide support and mentorship to those physicians who performed the procedure less frequently. For example, one nurse remembered supporting a physician through his first provision:

*What struck me about that day was my physician colleague, how his hands were shaking. And I remember putting my hand on his shoulder and just kind of nodding because we were there together and he had never done this before but we had spent a lot of time together previously. P1*

In the latter part of this quote, the nurse acknowledges that it was her previous relationship with the physician that allowed her to support him better. These supportive relationships within the MAiD team were acknowledged as an integral part of the process of a successful MAiD provision. Relationships facilitated the ability to know how each person would respond to such an impactful event, the ability for the nurse to step in and troubleshoot without offending the physician, and the ability to effectively debrief after the process. For example, this nurse described an experience of working with a physician who was unwittingly excluding the family’s access to the patient at the last moment, but she did not feel that she and the physician had enough of an established relationship for her to correct him:

*She [the client] was turned towards him [the physician] and her family was at her back and I thought what a shame that we couldn't take a moment and turn her to her family. But that was the first time I'd worked with that clinician. I didn't have any relationship with him at all. P2*

Another participant spoke of supporting a physician new to the MAiD process who was concerned that the patient had not died after administering the medication. Even though the nurse was certain that the patient had died, she took the stethoscope and listened for the heartbeat for a prolonged period of time so that the physician would be reassured. Such examples told a compelling story of the need for mutual support throughout the process of MAiD provision.

Participants also reflected on who might be excluded from the team, but who would nevertheless be deeply impacted by a MAiD death. For example, intravenous (IV) team members play an important role in the establishment and maintenance of the IVs upon which the success of MAiD administration rests. IV team members often establish the IV many hours before the provision to ensure that it is ready. During this insertion they often visit with patients and hear their story. As one nurse described it, you don’t put in the IV before you establish the relationship. But, even though these IV team members had established a relationship with the client, they did not have the team support when the client went on to receive MAiD. This nurse described encountering one such IV nurse. “*I remember an IV nurse starting an IV and for some reason she was waiting outside the door, the door was closed. I can’t remember exactly why but she was crying and I comforted her but, you know, she does not get the support we get on the unit*.” P6.

Privacy issues attaching to disclosure of a MAiD death also influenced who received support as part of the team. Home care nurses, acute care nurses, and residential care aids and nurses were frequently left out of the process for privacy reasons. Home care nurses described caring for long term clients who were not imminently dying and then being notified that they had suddenly passed away. The nurse would then follow up with the family and would be told that the client had received MAiD. It was not uncommon for these nurses to wonder why patients and families had not discussed this option with them, particularly in light of their long-term relationships. Nurses in general experienced this as being left out of the loop and, in some cases, it changed their practice in relation to MAiD:

*We had no idea they [clients and family] were thinking about it or mentioning it and no one had a clue and we’d just get notified that they’d passed away, which was really bizarre in the beginning. So, I think that was a turning point for me to make sure they knew all of their options and that they felt safe discussing all of their health with me. And no matter what they chose, they had those options on the table and that they could feel supported through the whole process if that’s what they chose. P12*

Stories from residential care were particularly challenging because of the close and enduring relationships that exist between clients and care aides. Clients might choose to keep their decision to access MAiD private, in part because they did not want to spend their last day saying goodbye or justifying their decisions. However, care aides were then taken by surprise by the death:

*Her request was not to tell any of the staff members until afterwards. Her care aides took that very poorly because they didn’t know. They were with her right to the last minute and it was a normal day. They took her to dinner, they took her out for a smoke, they took her back to her room. But then, they were told that she had died. P28*

So, while teamwork was considered the ideal of care, many were left out of the team for various reasons, and as a result did not receive the supports that those who were directly involved in the MAiD team experienced. Further, because MAiD was an impactful experience, those who had learned to work well together formed strong teams that were difficult for others to break into. For example, much of the MAiD referral process across Canada involves a centralized coordinator, often a nurse, who then assigns the patient to willing assessors and providers. These willing individuals are often the ‘go to’ people who work well together. As a result, others who would like to develop experience with the MAiD process may be inadvertently excluded, as was the case of the following participant:

*So, you have these pairs of teams and I think it speaks to the powerfulness of the experience. You need to work with a team that you're trusting in. Right? But there's an interesting sort of dynamic with that because, if you're a primary provider and you have a secondary person you use all the time, then you're just going to ask your secondary person. P2*

In summary, participants cited the importance of teamwork both to support a seamless MAiD process and to support those involved in this impactful experience. However, the ability to work within a team where relationships were well established had benefits beyond mutual support. It also facilitated the seamless organization of what was potentially a highly complex process.

### Processes: patient-centered aspirations in a complex system

Participants in this study described the complexity of facilitating a MAiD-related death. This complexity developed, in part, from the desire for a patient-oriented process. Participants recognized that MAiD would be the final act of healthcare they would perform for a client and that it would occur in a client’s last moment of life. This led to an intense desire to get the MAiD process ‘right’ and to provide the most person-centered care in the limited time that clients had left. For example, one nurse contrasted her previous practice in hospital to her current practice in MAiD using the analogy of a wheel and spokes. In her hospital practice she was the wheel and her patients were the spokes; in her MAiD practice that was reversed. However, this was a difficult aspiration to accomplish within a system that was generally not oriented toward providing patient-centered care. The achievement of such a patient-oriented perspective was plagued by difficulties.

This patient-centered perspective meant that nurses prioritized a MAiD-related request and/or provision over other duties. “*I will be dropping everything else that I’m doing when we have a MAiD case. It doesn’t matter what other priorities we have on the go, and I have lots of priorities because I’m the practice lead for a few areas.” P2* Priority tasks in a MAiD situation included assessing clients in a timely manner, coaching and educating clients and their families through the decision-making process, and most importantly, organizing a time and space for death in accordance with patient wishes. In some cases, this prioritization was driven by health policies that stipulated that patient requests had to be addressed within a specified time frame (typically a short one). In other cases, it was driven by the urgency of the request because clients were at risk of becoming incapacitated and then would not be able to provide the requisite final consent.

Once a request for MAiD had been initiated, nurses had to perform these priority tasks within systems that were organized to accommodate MAiD to varying degrees. This rural nurse spoke of the disruptions of continuity of care of caused by the MAiD care system. *“The client goes to their doctor, he refers to the MAiD steering committee, and I don’t know who those people are, they refer to my supervisor and it comes back to me. This is probably a patient that I already know.”* P39 In contrast, a seamless system included the presence of an organized referral system, willing MAiD assessors and providers, continuity of care with the existing system, ready access to MAiD-related paperwork and patient records, and a physical space within which to provide MAiD. However, even with all of these factors in place, the system could quickly become overwhelmed when a number of patients were requesting MAiD at the same time. For example, many patients and physicians preferred to schedule the death in the evenings or on weekends. This could prove challenging for MAiD providers, particularly for those who were engaged in MAiD as part of their regular Monday to Friday workload. One nurse described how she eventually had to set boundaries around her time. Even though she was supportive of MAiD, and was committed to its accessibility, she admitted that she did not want to spend all of her weekends providing MAiD.

Nurses also became overwhelmed when they were the only providers willing to engage in MAiD. One nurse practitioner shared that she had become the ‘go to’ person because the physicians in her community were not willing to perform MAiD. She was not sure whether this was because of moral reasons or a lack of adequate financial remuneration. But, she was quickly coming to the end of her emotional resources as a sole provider with limited support.

Participants also described having difficulty accessing patient records for their assessment process. This was particularly challenging when requests were urgent or when assessments were conducted over holidays. Further, there was little agreement about the amount of background information that should be provided to, or shared between, independent assessors. Physical space in which to provide MAiD could also be challenging, particularly for those patients who chose not to have MAiD performed in their home. In some cases, institutions where MAiD could occur (e.g., hospital, residence, or hospice) had policies that prohibited patient admissions that were solely for MAiD; however, nurses suggested that this accessibility was improving.

The ability to negotiate responsibilities was also an important part of system capacity. This was particularly relevant when there were dedicated MAiD teams. For example, one participant described how challenging it could be to decide whether a client should be referred to social work or to the MAiD team if a client expressed a wish for a hastened death. This was particularly the case if nurses did not have the time for the in-depth conversation that would enable them to better understand the intent of the request. Without this understanding, it was risky to do an immediate referral if the client was seeking support and it was risky to not do an immediate referral if it could be interpreted as limiting accessibility. Once a MAiD referral was made, it could be difficult to distinguish between the care responsibilities of regular providers and MAiD providers:

*So, it’s been a bit of a challenge to delineate what we’re doing in relationship to the request for assisted dying and what normal care still continues to be. So, that’s just a lot of conversations and we go and we meet with teams to say this is our bucket and this is your bucket and we’re all playing in the same sandbox to support the patient, but we all need to help each other. P3*

Once a MAiD request had been confirmed, and a time set for provision, nurses were also responsible to organize the support individuals, such as the IV team or pharmacists. In some cases, the IV team required 24 h advance notice to accommodate workload and conscientious objectors. Throughout this process, participants were confronted with complex organizational tasks, within systems that supported those tasks to varying degrees, and with the expectation that they would do their very best for this patient’s final hours.

Specific legislative requirements added a further layer of complexity to the system. For example, the legislation requires that one of the two MAiD assessors must also be the MAiD provider. However, in a person-centered approach, patients can indefinitely prolong the time between assessment and provision. This delay can have a number of implications. It might mean that the client presentation changes since the initial assessment, as described by this participant:

*About a month after I saw her for a secondary assessment I realized I've now become her primary provider. But because it's been so long I don't know whether to sign the Form C (clinician assessment form) or not because she actually doesn't have intolerable suffering at this point in time. She goes out for lunch every day with her friends like she's always done. But what happens if next week things turn upside down for her? Somebody else is going to have to come in and do the whole secondary assessment again. You know, without a system in place, it just makes things like that complicated that don't need to be complicated. P30*

The participant in the quote above found herself becoming the provider rather than the secondary assessor, but at least she was involved in both. In other situations, nurse practitioners were expected to be providers when they had not completed either of the original two required assessments. This usually occurred when there was a long delay between assessment and provision and the original assessors were no longer available. This placed these nurses in a difficult position, particularly if their assessment differed from the original assessment.

An additional legislative complexity involved the paperwork associated with a MAiD death as well as the coroner interview required post-MAiD in some jurisdictions. This paperwork became more complex with the new reporting requirements introduced by Health Canada in 2018. Nurses described having to endure these reporting requirements right after an impactful and exhausting MAiD administration. The most troubling aspect of these new reporting requirements was the need to defend one’s actions, similar to what one might do in a court of law. The legality of their participation was in question as described by this participant:

*The sense is that we have to prove that what we did was okay and that it was right. Our fear is that they're going to challenge us or ask a question that we won't have an answer to. That will put us in a position of feeling like, "Uh oh. What did I do now?" The new legislation [reporting requirements], make it worse. P30*

This same participant went on to describe the ironic nature of the self-reporting process that entailed grading one’s diligence in following the legislation:

*It is a three-page table that documents in a grid format how we're going to get into trouble if we do things wrong. I mean, that blows my mind, to be honest. I'm thinking, "Is this really necessary? I'm not planning on doing anything wrong (laughs). Why do you have to grade it?" It is a bit bizarre, you know. Not having done due diligence for foreseeable death is a score of 4 which means you get reported to your college. There are some 5s that mean you get reported to the police. But you think, "Hmm, okay. So, if I have provided this service to someone who shouldn't have qualified under the law and who wasn't actually dying then I essentially killed them. That's reported to my college? That should be murder, right? P30*

In this anecdote, the participant shows her struggle with the rules of a complex legislated and reporting process that determines the line between assisted death and murder and takes little account of her moral commitment to doing the right thing.

For nurses, the end result of trying to accomplish impactful patient-centered care within a complex system was excessive workload and emotional burden. This resulted in some nurses setting boundaries around their MAiD practice:

*I don’t find the provisions so emotionally draining, but it’s more the logistics and it’s a lot of work. The logistics of filling in 16 pieces of paper and making sure they’re all correct so you don’t get into trouble because the consequences are pretty significant. Then there’s organizing the pharmacy, going to pick up the medication, and organizing with the family, organizing with the nurse. Like, there is so much that goes into it. And that part can be so draining. And making it all happens as it should, you know, so that everything lines up. So, I think it’s important not to do too many cases. And that’s what I’ve been focusing on, making sure I’m not taking too much on. P25*

This nurse was choosing to set limits on her MAiD-related practice. But for other participants, the cost of working within a system that did not adequately support them was simply too much. The risks of not providing good care or of running afoul of the legal system were just too great.

*“Working in this haphazard framework you worry that patients are going to fall through the cracks because, you know, we all have busy worlds. Half of the practitioners I work with on an every-other-day basis say they’re not going to do this anymore.” P30.*

## Discussion

Findings from this study describe the impact of a legislated approach to assisted death on Canadian nursing practice and nurses’ experience. Such findings illustrate the proverbial rock and hard place in which nurses’ have obligations in relation to the MAiD legislation but find themselves in the complex situation of trying to negotiate best practices with variable support. Nurses in this study described a high degree of variability in policies and procedures, system processes, and team support across Canadian jurisdictions. They further described the importance of teamwork in facilitating such an impactful event. Finally, they described the complexity of facilitating a patient-centered death within a system that was not always well structured to support their efforts. These factors influenced their experiences with assisted death, and their willingness to take part, beyond any considerations of conscientious objection.

In discussing these findings, it is important to remember the limitations of this study. This was a qualitative study that explored the experiences of 59 registered nurses and nurse practitioners. This data was gathered just 2 years post legislation. As such, it represents nurses’ early experiences with MAiD and complements other early studies of nurses’ experiences in the Canadian context [17, 18]. Further, these interviews were conducted by telephone rather than in person. However, in reflecting on the richness, depth, and variability of participant responses, conducting these interviews by telephone may have provided a layer of necessary anonymity for such a controversial topic.

As discussed in the introduction to this paper, the legislated approach to MAiD requires *delegation, precision, and obligation* [5]. In this discussion, we will first explore some of the reasons for the variability of practice supports described in this study and relate those to the way that healthcare responsibilities are delegated in Canada. We will then discuss how adequate practice supports are essential to nurses’ abilities to meet the requirements of precision under the MAiD legislation. Finally, we will highlight the tensions that arise in nursing practice as a result of particular obligations inherent in the legislation.

### Delegation: supports as a reflection of sociocultural context

In a country that has chosen a ‘hard’ [5] approach to MAiD, it is intriguing that the development of nursing practice supports have been so variable across the country. In Canada, responsibility for healthcare rests with the provinces and territories. Provincial and territorial governments in turn delegate this responsibility to health authorities through policy direction and financing. Provinces and territories have structured their health authorities differently; some have one health authority for the entire province (e.g., Alberta and Manitoba), others have multiple authorities within a province (e.g., British Columbia and Newfoundland). Health authorities are designed, in part, to be responsive to the needs of their particular population [19]. Having multiple health authorities across the country can be inefficient when a task as complex as generating MAiD policies and supports is required. However, the variability in available practice supports that was described in these findings may also be an artifact of the interaction between the sociocultural context of each region and the nature of MAiD.

A number of factors make MAiD a contentious healthcare policy issue. First, MAiD is unique in its healthcare outcome. The intent of MAiD, unlike any other procedure done in healthcare, is always to definitively produce death [20]. Second, MAiD is a morally contentious act. Canadians have a range of responses to MAiD, from believing it to be a morally repugnant act to believing it to be a deeply compassionate act to relieve suffering [21]. Third, MAiD is new to Canada. Even though healthcare providers have always received requests to hasten death, only now do they have the legal authority to do so [22, 23]. Further, the experience of the death itself is vastly different from a normal death [8]. Such a different, morally complex, and new procedure is likely to be negotiated in profoundly different ways depending upon the sociocultural context. Provinces and territories are known to each be unique sociocultural contexts that are ultimately reflected in their healthcare policy and practice. Provinces in which the majority of citizens would reject MAiD as an option, may also be less likely to prioritize the implementation of MAiD-related structures. As important as it is to reflect the unique values and beliefs of the individuals of a particular region, nurses and other healthcare providers can also be placed in a challenging position. Canadians in certain clinical circumstances can claim a legal right to assisted death*,* but nurses residing within some jurisdictions may not have adequate systems in place to support their practice in fulfilling that right.

### Precision: practice supports in a legislated context

A legislated approach requires a high degree of precision, or a *“precise, specific, clear rule for every practice.”* (5 p.14) Our review of Canadian nursing regulatory documents indicated that these rules are found in legislation; regulatory guidelines; professional liability guidelines; and employer standards, guidelines, and policies [13]. Findings from this current study indicated that the necessary rules were present in some contexts but notably absent in others. In addition to these rules, participants spoke of a need for practice supports that would enable them to fulfill the requirements and obligations associated with a MAiD death. They understood that specific rules can only be enacted properly within a context of adequate support and, more importantly, when there was a mismatch between the required precision and contextual supports, nurses recognized that their practice was at risk. For example, the reporting guidelines required by Health Canada [24] were precise and specific in how they evaluated whether a MAiD procedure complied with the law. But, nurses at times perceived that they did not have adequate resources to meet those requirements. This was evident when nurses indicated they did not have access to required palliative care, to a supportive team, to policies, procedures and systems that would guide their practice, or to a sufficient number of assessors and providers to support the number of patients seeking MAiD. A perceived lack of a supportive system put some nurses in an untenable position. They were engaging in a high-risk practice that contained precise criteria to differentiate between “*MAiD and murder.*” But they were doing so within what one nurse described as “*haphazard*” systems that do not support the necessary degree of precision. Further, it is important to note that while there are reporting systems in place in Canada, there is no specific oversight and review of MAiD practices. This makes it difficult for practitioners to benchmark good practice other than through their own self-report. Also, there is little data upon which to further develop national policies and best practices. The result was that some practitioners were choosing to limit their involvement in MAiD or refuse to engage in it altogether. However, it is important to remember that, when these practice supports were in place, nurses who participated in this study felt confident in their ability to meet precision requirements.

Not all of the uncertainty in this study was related to a lack of precision at the healthcare policy level. Some related this lack of precision to ambiguity in the legislative language itself. Of particular concern is the definition given in *Bill C-14* to “grievous and irremediable medical condition,” one of the eligibility requirements for MAiD. A “grievous and irremediable medical condition” is defined within *Bill C-14* as requiring four criteria to be met, two of which are that death has become “reasonably foreseeable” and that the “illness, disease or disability or state of decline is causing enduring and intolerable physical or psychological suffering.” The concepts of “reasonably foreseeable” death and “intolerable suffering” in particular have been the subject of significant and ongoing controversy in healthcare, legal and patient advocacy communities [25–27]. Indeed, since, Bill C-14, the Quebec Superior Court has struck down the requirement that death be “reasonably foreseeable.” [28] Such controversy was reflected within this study as well. Nurses reported that a reasonably foreseeable death was being interpreted differently by different clinicians. Documents that have attempted to clarify this language from a legal perspective (e.g., 26) may not necessarily be congruent with clinicians’ clinical and moral judgement. This may explain some of the findings in which nurses felt that physicians were placing access barriers in front of patients seeking MAiD. What nurses interpreted as physicians limiting accessibility by telling patients they were not ready for MAiD yet, may actually have been a physician interpretation that the patient’s death was not yet *foreseeable*. If the death was not foreseeable, then provision of MAiD would violate the legislation and render the assisted death a criminal act. Such lack of precision in terminology led to divergent opinions and practice, and ultimately tensions, among clinicians.

In terms of the language of “irremediable suffering,” nurses in this study believed that such suffering could only be defined by the patient. However, this created doubt in their abilities to adhere to the legislation. This was evident in the difficulty experienced by a participant in checking the box on the reporting guidelines to confirm that the client was enduring irremediable suffering while knowing that the client was still participating in daily enjoyable activities. Such leeway in interpretation made it difficult for nurses in this study to feel as though they were fulfilling their obligation to practice within clear and specific rules. This uncertainty was compounded when nurses could not draw upon the collective wisdom of a supportive team.

### Obligation: accessibility and participation

A legislative approach to assisted death also implies a high degree of obligation. Normally, this implies an obligation to fulfill rules in a precise manner [5]. But the ideal of obligation has taken on a new dimension within Canada because the MAiD legislation was developed because of an appeal to *Charter of Rights and Freedoms* guarantees. As such, MAiD in Canada has been framed as a right, and as a right, it brings issues of accessibility to the fore. If Canadians have a legal right to MAiD, then the healthcare system has a responsibility to make MAiD accessible (particularly given the *Canada Health Act*, which confirms accessibility as one of the five essential conditions of the Canadian health system). This idea has generated much debate in Canada. Some MAiD proponents have argued that accessibility means that MAiD should be discussed alongside other end-of-life options, even if the patient has not specifically requested information about MAiD [29]. Accessibility to MAiD may also be challenging in rural and remote areas where there may be few providers willing to provide MAiD, and where taking on MAiD-related responsibilities can have significant implications for those rural practitioners who also provide palliative care [3, 30]. This is particularly difficult when many parts of rural and remote Canada still do not have access to good palliative care [31]. Gaps in accessibility to palliative care explain, in part, data in this study about how palliative care providers have resisted the development of MAiD. Some practitioners have expressed their concern that the philosophies of assisted dying and palliative care are incompatible with one another while others have argued that the two philosophies may not actually be contradictory [32, 33]. However, if the political will to provide accessibility to palliative care is not as strong as the political will to provide accessibility to MAiD, then inevitably it will be easier for patients to access MAiD than to access palliative care. This is of even greater concern when one considers the potential end-of-life healthcare cost savings generated by MAiD-related deaths in Canada [34].

The obligation to make MAiD accessible influenced nurses’ experiences both positively and negatively. Some nurses worked within well-resourced teams dedicated to patient-centered access, thus fulfilling their ideal of MAiD access for patients who wanted it. However, facilitating accessibility could be more problematic outside of such a team structure. Those nurses who felt that they were obligated to provide MAiD because others in their community refused to do so, found themselves in a difficult position. This was evident in the data when nurses had to erect boundaries around their involvement, either because they were trying to organize this precise act within a poorly designed system or because they were experiencing ill effects of trying to do this emotionally laborious task alone. But, in a climate that focusses on an obligation to access, it is difficult for nurses to decline to participate, particularly as employees of healthcare. For example, in this study, once a decision-maker had chosen to embed a MAiD role within a particular nursing role to improve access, it then became difficult for nurses to decline to participate in MAiD and still fulfill their employment obligations. A number of nurses in this study reflected on how they had never imagined that they would be asked to participate in such an act within their nursing career. Notably, in this study, nurses’ decision to not participate was not always because of a conscientious objection. Rather, that decision could often be attributed to a lack of resources and support, to a difference in philosophies (e.g., palliative care and MAiD), or to a belief that MAiD was inappropriately overshadowing other important healthcare priorities. These reasons for non-participation have been discussed in the literature related to institutional participation in MAiD [35]. In these situations, and in situations where nurses were conscientious objectors, nurses’ experiences were influenced by how responsive and respectful leaders were in accommodating their decision of whether or not to participate in MAiD.

### Clinical implications

MAiD legislation in Canada has led to a dramatically new form of practice There is an opportunity to unpack multiple layers of nursing practice experience to better understand both the implications of the structural context of practice and the moral impact of various care settings and teamwork arrangements. Findings of this study demonstrate the powerful impact of organizational leadership on the workplace policies and culture that significantly determine how nurses experience this new care option. We can also see how the potentially conflicting worldviews of different practice sectors, in this case the specialist palliative care sector and the sector involved in MAiD provision, not only shape the care options accessible to patients, but also the nuances of nursing engagement with patients who are considering or completing MAiD. These data demonstrate a full range of care cultures, from those that place all concerned in states of extreme tension to those that create space for the ambiguity and complexity characteristic of MAiD at this time in Canadian history. As more and more nurses across the international context encounter patients for whom MAiD is a possibility, it will be increasingly important that procedures and supports be put in place to support nursing practice. This is particularly essential where a legislated, or hard, approach to assisted death requires precision, obligation, and delegation. Further, robust policies, and perhaps more importantly, supportive procedures, are required to ensure that nurses can choose to participate, or not, in this radical new end of life care option. Those who choose to participate require supported practice; those who choose not to participate need the freedom to do so without fear that it will negatively influence their colleagues or their employment options. This will be particularly relevant in international contexts where assisted death becomes embedded within health systems, similar to how it has been enacted in Canada.

The nature of the Canadian legislation has spawned new and intimate practice teams that support practitioners to provide patient-centered, high quality care, and mutual support during such a momentous time. However, these data also reveal the potential disruption of currently existing teams and a lack of recognition of the supportive work done by those who may not be directly involved in MAiD assessment and provision. It further reveals the difficulties encountered by those who act as MAiD assessors and providers without the presence of a supportive team. So, although the MAiD legislation provides specificity as to the roles and obligations of assessors and providers, such work cannot be solely delegated to these individuals. Comprehensive care must consider the many collateral persons who provide support throughout the care trajectory, from the time patients first consider MAiD through to the stage of bereavement. All members of the care team clearly feel the need for guidance and insight as to how to manage the moral tensions associated with providing the best care possible through to the end. Further, members of these teams require expertise in how to assess and negotiate the complex patient request for death that may or may not reflect and request for MAiD. Belgian law stipulates that the nursing team should be consulted regarding patient euthanasia requests, although no such requirement exists in the Netherlands [12]. Evidence derived from nurses in Belgium has attested to the complexity of these conversations. (7, 8) Countries considering the legalization of assisted death should carefully consider the impact of teamwork on best practices, including those having to do with communicating with patients and families.

## Conclusion

These findings have permitted a glimpse into the morally difficult and organizationally complex work that a legislated approach to MAiD places upon nurses who are already often coping with highly challenging work environments. Variable practice supports, leadership philosophies, team structures, and system and legislative supports greatly influenced whether nurses were able to confidently meet the hard requirements of a legislated approach. Clearly, in light of a legislated approach to MAiD that requires high degrees of delegation, precision, and obligation we have much work to do in supporting nursing through basic and continuing educational programming, care pathways and best practice guidelines, and workplace teams and environments. Further, we must continue to try to understand the important lessons that the experience of nurses of being caught “between a rock and a hard place” can offer with respect to what this radical new care options mean for all concerned.

## Supplementary information

**Additional file 1.** Interview guide, Policy, Practice, and Ethical Implications of Medical Assistance in Dying: Semi-Structured Interview Guide. This was the initial guide that received ethical approval; however, it is important to note that this was a draft guide. In keeping with semi-structured interviews, additional questions may have been asked.

## Data Availability

The datasets generated and/or analysed during the current study are not publicly available due to participant confidentiality.

## Abbreviations

CNA: Canadian Nurses Association
MAiD: Medical Assistance in Dying
