# Supplementary material for: The rocks and hard places of MAiD: a qualitative study of nursing practice in the context of legislated assisted death
Source: BMC Nurs. 2020 Feb 17;19:12. doi: 10.1186/s12912-020-0404-5 (PMC7025406; doi:10.1186/s12912-020-0404-5)
Supplement: Supplementary file 1 — Additional file 1. Interview guide, Policy, Practice, and Ethical Implications of Medical Assistance in Dying: Semi-Structured Interview Guide. This was the initial guide that received ethical approval; however, it is important to note that this was a draft guide. In keeping with semi-structured interviews, additional questions may have been asked. [file 12912_2020_404_MOESM1_ESM.docx]

Practice, Policy, and Ethical Implications of Canadian Nursing Roles in Medical Aid in Dying (MAiD)

Please begin by telling us about your involvement with MAiD to date.

**Schedule A: For those who have had some degree of involvement with MAiD.**

Policy and practice supports. *We first want to inquire a bit about how MAiD is enacted in your context and what supports you have available to assist you.*

1. Can you tell us how the process of MAiD tends to occur in your practice context? What is your role in relation to that process? Do you have a MAiD team?
2. What resources and practice supports are available to assist you in caring for MAiD patients? How do you use those practice supports? What practice supports are missing in your opinion?
3. How would you describe your familiarity with the regulations that govern your practice in MAiD?
4. How would you describe your knowledge related to MAiD, the MAiD process in general, and your role in the MAiD process specifically? How confident do you feel in caring for MAiD patients? What has helped or hindered that confidence?
5. Can you describe how MAiD fits or does not fit with palliative care in your context?

Personal experiences*:*

*Now we want to focus a bit more on your experiences with MAiD.*

1. How did you decide to participate in MAiD?
2. Can you help us to understand what it is like to communicate about MAiD in the current healthcare climate?
3. What is it like to have conversations with others about MAiD
4. Can you describe the first time you cared for a MAiD patient? What was that like for you?
5. Can you help us to understand what it is like to work with patients who are in that initial stages of considering MAiD? What have you learned about handling those initial inquiries?
6. Those receiving MAiD in Canada have to meet certain eligibility criteria. Can you help us to understand how these terms are being defined in your practice?
7. There are a number of safeguards that must be met for a patient to receive MAiD (e.g., criteria, consent, waiting times, etc.). As part of your practice related to MAiD, do you review those safeguards as they relate to particular patients? If so, how confident are you that they are being met consistently?
8. What have you learned about good nursing care during the MAiD process?
9. How do you understand your nursing scope of practice in relation to MAiD? When you think about your regulated nursing scope of practice in relation to MAiD, how does that scope match your realities of everyday practice?
10. As you think about your practice in MAiD are there issues that cause you concern or uncertainty? If so, what are they?
11. When you think about your experiences with MAiD overall, what do you see as the positives and what are the challenges? Another way to think about this might be to describe a case that went really well and one that you have struggled with.
12. What have we forgotten to ask about that you think we should ask?

**Schedule B: For those who have been a conscientious objector.**

Policy and practice supports. *We first want to inquire a bit about how MAiD is enacted in your context and what supports you have available to assist you.*

1. Can you tell us how the process of MAiD tends to occur in your practice context?
2. What resources and practice supports are available in your practice context? Which have been specific to your ability/decision to not participate in MAiD?
3. How would you describe your familiarity with the regulations that govern your practice in MAiD?
4. How would you describe your knowledge related to MAiD, the MAiD process, the nursing role in MAiD, the role of other team members in the MAiD process?
5. Can you describe how MAiD fits or does not fit with palliative care in your context?

Personal experiences*: Now we want to focus a bit more on your experience of choosing not to participate in MAiD.*

1. How did you decide not to participate in MAiD? Were there challenges in coming to that decision? If so, what were they, and how did you resolve them? Are there any aspects of MAiD you are willing to be involved in?
2. Can you help us to understand what it is like to communicate about MAiD in the current healthcare climate.
3. What is it like to have conversations with others about MAiD?
4. Can you describe the first time you cared for a MAiD patient for whom you chose not to participate? What was that like for you? At what point did your involvement with the patient cease? Did any involvement in care continue?
5. Can you help us to understand what it is like to work with patients who approach you to learn more about MAiD? What have you learned about handling those initial inquiries?
6. Those receiving MAiD in Canada have to meet certain eligibility criteria. Can you help us to understand how these terms are being defined in your practice?
7. There are a number of safeguards that must be met for a patient to receive MAiD (e.g., criteria, consent, waiting times etc.).When you have a patient who chooses to receive MAiD, do you review those safeguards as they relate to your patients? If so, how confident are you that they are being met consistently?
8. What have you learned about good nursing care during the MAiD process for nurses who choose not to participate? What about once MAiD is completed?
9. As you think about MAiD in your workplace, are there issues that cause you concern or uncertainty? If so, what are they?
10. When you think about your experiences with MAiD overall, what do you see as the positives and what are the challenges?
11. What have we forgotten to ask about that you think we should ask?
